# Comparative Severity Assessment of Genetic, Stress-Based, and Pharmacological Mouse Models of Depression

**DOI:** 10.3389/fnbeh.2022.908366

**Published:** 2022-06-16

**Authors:** Anne Stephanie Mallien, Natascha Pfeiffer, Christiane Brandwein, Dragos Inta, Rolf Sprengel, Rupert Palme, Steven R. Talbot, Peter Gass

**Affiliations:** ^1^Research Group (RG) Animal Models in Psychiatry, Department of Psychiatry and Psychotherapy, Medical Faculty Mannheim, Central Institute of Mental Health, Heidelberg University, Heidelberg, Germany; ^2^Department for Community Health, Faculty of Natural Sciences and Medicine, University of Fribourg, Fribourg, Switzerland; ^3^Max Planck Institute for Medical Research (MPIMF), Heidelberg, Germany; ^4^Department of Biomedical Sciences, University of Veterinary Medicine, Vienna, Austria; ^5^Institute for Laboratory Animal Science, Hannover Medical School, Hanover, Germany

**Keywords:** depression model, mouse, severity assessment, Laboratory Animal Science, 3R, GluA1 (AMPA receptor subunit GluR1), stress, fecal corticosterone metabolites

## Abstract

The use of animals in neurosciences is pivotal to gaining insights into complex functions and dysfunctions of behavior. For example, various forms of physical and/or psychological stress are inherent to various animal models for psychiatric disorders, e.g., depression. Regarding animal welfare, it would be mandatory to use models that inflict the least amount of stress necessary to address the underlying scientific question. This study compared the severity of different approaches to induce depression in mice: mutagenesis in GluA1 knockout, immobilization stress, and stress-induction *via* stress hormone treatment. While genetic alterations potentially represent a lifelong burden, the temporary intervention only affects the animals for a limited time. Therefore, we used home cage-based behavioral and physiological parameters, including nest building, burrowing, body weight, and fecal corticosterone metabolites, to determine the well-being of male and female mice. In addition, we performed an evidence-based estimate of severity using a composite score for relative severity assessment (RELSA) with this data. We found that even though restraint stress and supplementation of corticosterone in the diet both aimed at depression-related precipitating stress effects, the latter affected the well-being much stronger, especially in females. Restraint leads to less noticeable well-being impairments but causes depression-associated anhedonic behavior. Mice of both sexes recovered well from the stress treatment. GluA1 KO and their littermates showed diminished well-being, comparable to the immobilization experiments. However, since this is a lifelong condition, this burden is not reversible and potentially accumulative. In line with the 3Rs (Replacement, Reduction, and Refinement), the process of choosing the most suitable model should ideally include an evidence-based severity assessment to be able to opt for the least severe alternative, which still induces the desired effect. Promoting refinement, in our study, this would be the restraint stress.

## Introduction

The use of animal models in neurosciences, especially in experimental psychiatry, is pivotal to gaining mechanistic insights into behavior’s complex functions and dysfunctions (Homberg et al., 2021). Comprehension of such mechanisms provides the basis for understanding critical aspects of pathogenesis, pathophysiology, and the development of new therapeutic approaches for psychiatric disorders. There is an undeniable necessity for further research in this area. However, since animal experiments and models are mainly based on mimicking the human condition, various forms of physical and/or psychological stress are inherent to a variety of psychiatric animal models, also because stress is an essential component of psychiatric diseases, in particular in affective disorders (Nestler and Hyman, 2010; Neumann et al., 2011).

The use of animals for research is legally regulated on the explicit understanding that such use will provide significant new insights facilitating relevant benefits. No unnecessary harm will be imposed on the animals. Strains and distress should be avoided or minimized. This ethical dilemma – to balance the gain of knowledge with potential harm to the animals – was the basis for the development of the “3R principle” and, more recently, for “harm-benefit-analyses” (Wurbel, 2017; Grimm et al., 2019). Meanwhile, these principles have been integrated into European legislation (Directive 2010/63/EU) and good scientific practice, e.g., in the ARRIVE Guidelines or the Basel Declaration (Kilkenny et al., 2010; Percie du Sert et al., 2020).

No generally accepted standardizing body is available for evidence-based assessment and classification of severity regarding maintenance, handling, and experimental procedures in laboratory animals, particularly rodents (Birnbacher, 2009). Thus, assignment to the severity grades “mild,” “moderate,” or “severe” in the EU Directive 2010/63 was not evidence – but rather eminence-based, from an anthropomorphic perspective. Rodents might have a different perception due to their species-specific adaptation to the environment. In principle, it is evident that stressors, e.g., foot shocks, social isolation, or restraint, cause considerable distress. But why is a transient state of distress, e.g., learned helplessness, regarded as severe and not as moderate (Mallien et al., 2020)? Do different forms of stress evoke different severity levels? Are behavioral strategies to model depression symptoms more severe than transgenic models with lifelong or induced mutations of candidate genes for a disease? And, Do these stressors, especially if transient, compromise the animals more than experimentally induced cancer or inflammation? These questions are all insufficiently or unanswered in the EU Directive.

In the present study, we used established behavioral and physiological indicators for impaired welfare for a comparative analysis of models for depression in mice. Moreover, our analysis included an algorithm-based comprehensive composite score to detect welfare impairments objectively and provide a relative severity assessment (RELSA) of the different modeling strategies (Talbot et al., 2020). We assessed the severity of several established models for depression based on targeted mutagenesis and stress-based treatments. All models have been shown to induce defined syndromes in mice, leading to alterations in the animals’ emotional or cognitive behaviors. *GluA1* knockout mice represent a well-known animal model for depression based on mutation of the AMPA receptor subunit 1 (Zamanillo et al., 1999; Bannerman et al., 2004; Chourbaji et al., 2008; Wiedholz et al., 2008; Sanderson et al., 2009, 2017; Fitzgerald et al., 2010; Inta et al., 2010; Bygrave et al., 2016, 2019; Eltokhi et al., 2020; Ang et al., 2021), which is consistent with the glutamate hypothesis of depression (Sanacora et al., 2012). As stress-based models, we used the daily experience of restraint stress on the one hand and on the other, chronic pharmacological treatment with the stress hormone corticosterone (Moda-Sava et al., 2019). We analyzed and compared the severity before, during, and after the respective stressor/treatment.

## Materials and Methods

### Experimental Design

We aimed to assess models of different modalities: one targeted mutagenesis line in Experiment 1 and two stress-based models in Experiment 2.

**FIGURE 1:**
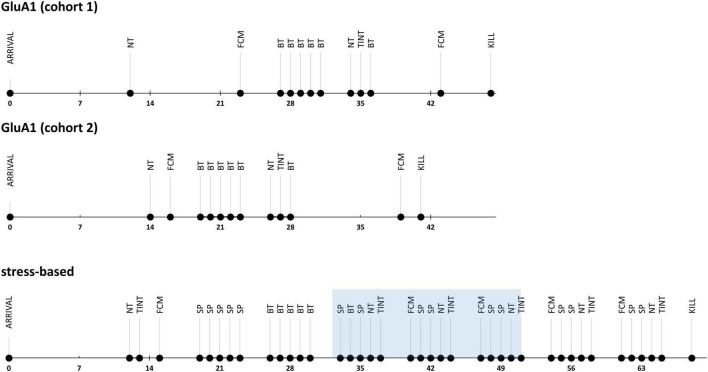
Experimental design: time lines of the severity assessment experiments. NT, nest test; FCM, fecal corticosterone metabolite sampling; BT, burrowing test; TINT, time to integrate into nest test, SP, saccharin preference.

In Experiment 1, we measured the severity-related parameters only once since the animals’ investigated genetic alterations are life-long conditions (Figure 1). In Experiment 2, we observed the severity in the stress-based models in a longitudinal approach to detect the exacerbation due to the treatment and the subsequent alleviation afterward (Figure 1). The experimenters were blinded for genotype and treatment during the behavioral experiments.

#### Animals

In Experiment 1, we investigated the genetically modified mouse line *Gria1^–/–^* (Zamanillo et al., 1999). The *Gria1* gene encodes the GluA1 subunit of the AMPA receptor. GluA1 KO mice were bred in the Interfacultary Biomedical Faculty (IBF, Heidelberg, Germany). We tested 11 male and 14 female GluA1 knockout mice and the respective 14 male and 11 female littermate controls in 2 cohorts. The mice were 7–10 weeks old at arrival at the Central Institute of Mental Health (CIMH, Mannheim, Germany).

For Experiment 2, we purchased 8-week-old male and female C57BL/6N mice from Charles River (Charles River Laboratories, Sulzfeld, Germany) and assigned 10 per sex to 3 different groups: *restrained*, *corticosterone*, and *control*. Saccharin preference (SP) and burrowing performances were used to distribute the mice into groups with equivalent baseline behaviors pseudorandomly.

All animals were single-housed in Type II cages with bedding (Abedd Espen MIDI, ABEDD, Vienna, Austria), cotton nestlet (Zoonlab, Castrop-Rauxel, Germany), and *ad libitum* access to food (LasQCdiet Rod16-H, Altromin, Soest, Germany) and tap water. The housings were temperature- (22 ± 1°C) and humidity-controlled (45 ± 5%) under 12:12 h dark:light cycle with lights off at 7 a.m. All procedures were approved by the German animal welfare authorities (35-9185-81-G-198-17, Regierungspräsidium Karlsruhe) and performed strictly according to the regulations of animal experimentation within the European Union (European Communities Council Directive 2010/63/EU).

#### Treatment

For the chronic corticosterone treatment (Moda-Sava et al., 2019), we diluted corticosterone in 100% ethanol and added water to receive a final concentration of 0.1 mg/ml corticosterone and 1% ethanol. This was supplied to the animals in their drinking bottle. Since two female mice reached the humane endpoint and were euthanized after 18 days, we decided to shorten the chronic treatment from the initially planned 21 days to only 18.

We adjusted the duration of repeated restraint accordingly, which was then performed for 18 days with physical restriction for 1 h per day in plastic tubes (26 mm diameter) with holes for breathing in a separate experimental room during the dark phase.

### Behavioral Analysis

#### Nesting

Nest building was evaluated according to a rating scale on the shape and cohesion of the nest as previously described (Deacon, 2006; Mallien et al., 2020). The mice were placed in a new home cage with a cotton nestlet 1 h before the onset of the dark phase, and the score was determined after 13 h. Subsequently, we performed the “Time to integrate into nest test” (TINT). For this, additional nesting material (sizzle, Zoonlab, Castrop-Rauxel, Germany) was introduced into the diagonally opposing corner of the nest site within the first 3 h of the light phase, and the latency to integrate the novel material into the existing nest site was observed for 10 min (Hager et al., 2015; Mallien et al., 2020). In the GluA1 study, we also performed a daily nest scoring, always at the same time of day [corresponding to the 13 h timing of the nest test (NT)].

#### Burrowing

To analyze the burrowing behavior, we placed bottles (14 cm long × 5.5 cm in diameter) filled with food pellets at the rear of the home cage 1 h before the dark phase and let the mice burrow as previously described (Sensini et al., 2020). The amount of burrowed material (% of total weight) was assessed after 6 h. All mice were accustomed to the procedure for five consecutive days before the final testing day.

#### Fecal Corticosterone Metabolites

Feces samples were collected for 24 h in a secondary home cage and were processed as described (Sensini et al., 2020). Briefly, an extract of dried and homogenized feces was produced with 80% methanol, and an aliquot was analyzed in a well-established and validated 5α-pregnane-3β,11β,21-triol-20-one enzyme immunoassay (EIA) (Touma et al., 2003, 2004; Palme, 2019; Sensini et al., 2020).

#### Saccharin Preference

We investigated the preference for saccharin solution of the stress-based models as previously described (Kronenberg et al., 2012; Klein et al., 2015; Boldt et al., 2021). Briefly, we determined the intake from two drinking bottles in 24 h on four consecutive days with either two bottles filled with water (day 1 and 3) or one with water and one with a 0.1% saccharin solution (day 2 and 4). Day 1 and 3 were used to detect putative side bias. On days 2 and 4, we determined the preference for saccharin.

### Statistical Analyses

We analyzed all outcome measures [nesting, burrowing, fecal corticosterone metabolites (FCMs), SP] with linear models. In Experiment 1, the dependent variables treatment, cohort, and sex were modeled as interactions (parameter ∼ treatment: cohort: sex). In Experiment 2, the dependent variables treatment, phase, and sex were modeled as interactions (parameter ∼ treatment: phase: sex). Factors were treated as simple treatment contrasts with the default level in the intercept. Subsequent multiple comparisons were tested with the Tukey–Kramer *post hoc* test. The loss of two highly burdened females might have distorted the results of the post-stress phase for the females. No animals were excluded from the study or any statistical analyses. The single animal served as an experimental unit.

We used the RELSA (Talbot et al., 2020) score to compare different models. This algorithm-based comprehensive composite score detects relative welfare impairments from multi-dimensional input parameters of physiology and behavior. We chose to include body weight (BW), burrowing performance, and fecal corticosterone metabolite concentrations as the most suitable parameters to determine the severity. RELSA achieved relative comparability by expressing the multi-dimensional change in the input variables’ differences regarding a reference set with a fixed severity quality. In this study, the reference set was obtained from values in the restrained experiment. In addition, the time-independent maximum RELSA score (RELSA_*max*_) in each animal was used to compare different experiments and subgroups quantitatively. Finally, we used the RELSA to present the state of severity over time (RELSA_*flow*_) or the most pronounced severity displayed by the animals throughout the procedure (RELSA_*max*_).

## Results

### Experiment 1: Severity Assessment of GluA1 KO Mice

#### Body Weight

**FIGURE 2:**
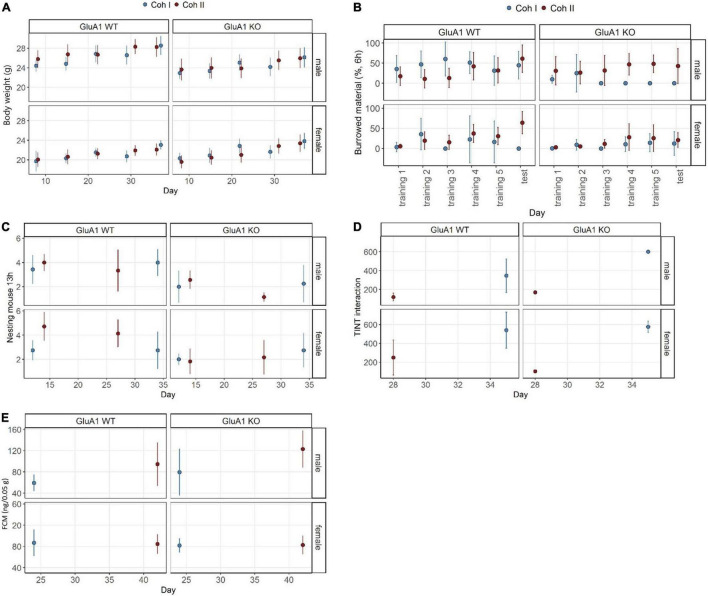
Well-being-associated parameters in GluA1 KO and WT. Several cohort and sex-specific differences became evident. **(A)** GluA1 KO males show reduced body weight. **(B)** GluA1 KO males burrow less than WT, but not consistent over both cohorts, while the females rarely perform at all. **(C)** Nest complexity is lower in GluA1 KO. **(D)** The cohort largely affects the time to integrate into nest test (TINT). **(E)** GluA1 KO males have higher baseline concentrations of fecal corticosterone metabolites (FCMs) in only one cohort. The concentration in females was comparable between cohorts and genotypes. Data was analyzed using linear models (lm) in R, GluA1 KO ♂ *n* = 11, ♀ *n* = 14, controls ♂ = 14, ♀ = 11, error bars are mean ± 95% CIs.

GluA1 KO and WT mice showed normal weight gain over time [time: *F*(1,234) = 109.98, *p* < 0.0001] and the typical sex differences with females weighing 5.19 (SE 0.54) g less than the intercept [β = 26.23 (SE 0.31) g, *p* < 0.0001] (Figure 2A). The mean weight of GluA1 KO was significantly reduced by 1.92 (SE 0.54) g (*p* = 0.0004). This reduction was mainly driven by differences between the male KO and WT mice [cohort I: male GluA1 WT – male GluA1 KO: β = 1.917 (SE 0.539) g, *p* = 0.0025; cohort II: male GluA1 WT – male GluA1 KO: β = 2.564 (SE 0.490) g, *p* < 0.0001], while no differences in BW became apparent for females of the different genotypes. This also led to a treatment: sex interaction [*F*(1,234) = 43.19, *p* < 0.0001]. No differences were detected between the cohorts.

#### Burrowing Test

The overall estimate of burrowed material in Experiment 1 was 45.09% (SE 4.19) (=intercept) (Figure 2B). The GluA1 KO led to a decrease of 39.25% (SE 7.26) (*p* < 0.0001) of burrowed material. Also, female mice burrowed 32.04% (SE 7.26) less. Female mice burrowed almost not at all and showed no significant differences due to treatment. The female GluA1 KO burrowed 7.84% (SE 4.19) and 15.81% (SE 4.84) (in cohorts I and II, respectively), while their controls burrowed 13.05% (SE 5.93) and 28.83% (SE 5.93). The maximum performance was detected in male controls with 45.09% (SE 4.19) and 29.52% (SE 4.84). The burrowing performance was higher in cohort II [cohort: *F*(1,252) = 5.4911, *p* = 0.0199], driven by the relatively high performance of male GluA1 KO mice with 37.95% (SE 4.48) estimated marginal means. The other GluA1 KO burrowed less: males cohort I 5.874% (SE 5.93); females cohort I 7.84% (SE 4.19); females cohort II 15.81% (SE 4.84).

#### Nest Test

The GluA1 KO mice built less complex nests with 1.61 (SE 0.50) (*p* = 0.0017) lower complexity scores compared to the intercept [β = 3.73 (SE 0.29), *p* < 0.0001]. No general sex-specific or cohort-dependent differences were found (Figure 2C). Nests of females scored 0.93 (SE 0.50) (*p* = 0.0505) less. Differences between male mice became obvious in the direct comparison: the KO scored lower 1.61 (SE 0.50) (*p* = 0.0089) in cohort I and 1.81 (SE 0.45) (*p* = 0.0006) in cohort II. In female mice, there were no differences between KO and WT performance in cohort I, but in cohort II the score of KO was 2.43 (SE 0.45) (*p* < 0.0001) lower.

#### Time to Integrate Into Nest Test

We found a general delay in time to integrate the material into the nest [genotype: *F*(1,26) = 10.6566, *p* = 0.003069], indicating a more hesitant behavior in GluA1 KO mice [253.75 (SE 122.42) s, *p* = 0.0482] compared to the intercept [β = 346.25 (SE 54.75) s, *p* < 0.0001] (Figure 2D). Female mice generally showed higher latencies [193.75 (SE 94.83) s, *p* = 0.0513]. However, we could not find severe differences in direct comparisons of estimated marginal means between the groups within the same cohort and same sex. There was an overall effect of sex [(sex: *F*(1,26) = 5.07, *p* = 0.0330], but similarly, no significant differences in direct comparisons.

#### Fecal Corticosterone Metabolite Sampling

Fecal corticosterone metabolite concentration of GluA1 KO mice tended to be increased [*F*(1,42) = 3.95, *p* = 0.0534] (Figure 2E). The intercept of FCM was 59.4 (SE 8.8) ng/0.05 g and was lower in GluA1 KO mice [20.2 (SE 15.2) ng/0.05 g, *p* = 0.1920]. In cohort I, the concentrations were significantly higher [63.6 (SE 12.9) ng/0.05 g, *p* = 0.0001] in male KO than in controls. This was neither found in male mice of cohort II nor in genotype comparisons between female mice. No sex effect was evident, but a cohort effect [cohort: *F*(1,62) = 9.81, *p* < 0.0032] was observed. The concentrations of cohort II were significantly decreased [β = 35.1 (SE 13.4) ng/0.05 g, *p* = 0.0122].

### Experiment 2: The Development of Severity in Stress-Based Models Over Time in Restrained, Corticosterone, and Control Mice

**FIGURE 3:**
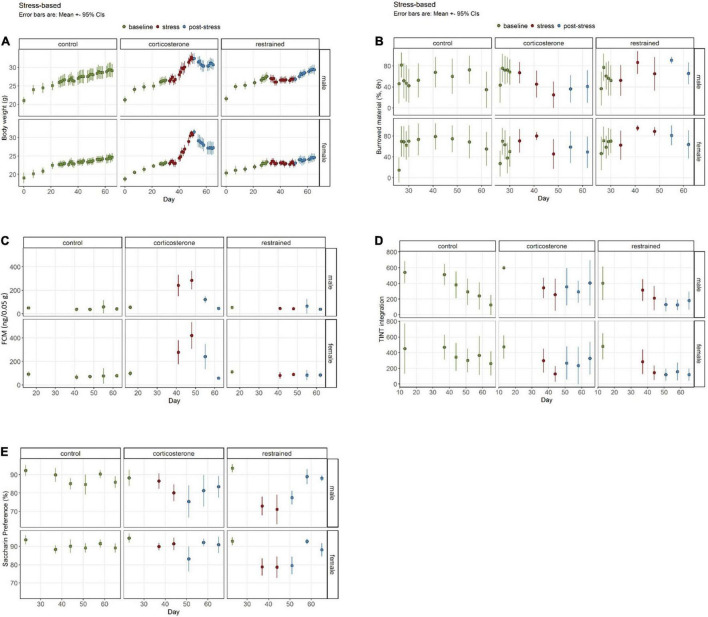
Well-being-associated parameters in stress-based models. **(A)** Corticosterone intake increases body weight, while restraint causes diminished growth. **(B)** The burrowing performance was not influenced by restraint, but by corticosterone treatment. **(C)** Increased concentrations of fecal corticosterone metabolites (FCMs) are evident in the corticosterone group but not in the restrained animals or controls. **(D)** The latency to integrate new nesting material into the nest site is elevated in the corticosterone group in the post-stress-phase. **(E)** Interestingly, only a small and sex-dependent decrease of saccharin preference in corticosterone-treated mice is observable, while the restrained mice demonstrate a stronger effect. Data was analyzed using mixed linear models, *n* = 10 per group, except ♀ corticosterone *n* = 8, error bars are mean ± 95% CIs.

We separated the data into three phases for this analysis: the initial baseline assessment, the stress phase during the treatment, and the post-stress evaluation (Figure 3).

#### Body Weight

For the BW we found the typical increase of weight over time [phase: *F*(2,1919) = 941.21, *p* < 0.0001] and the sex differences [sex: *F*(1,1919) = 2717.93, *p* < 0.0001] (Figure 3A). The intercept was 66.4 (SE 5.1) g (*p* < 0.0001) and was 0.7 (0.3) g (*p* = 0.0251) increased in restrained mice. Females weighed 3.2 (0.3) g (*p* < 0.0001) less than the intercept. In the males, we found no significant differences in the estimated marginal means during the baseline phase between the restrained group with 25.9 (SE 0.2) g and the corticosterone group with 25.2 (SE 0.2) g. During the stress phase, the mean BW of *corticosterone* mice increased significantly by 4.0 (SE 0.3) g (*p* < 0.0001). The mean BW of *restrained* male mice barely increased by only 0.8 (SE 0.3) g (*p* = 0.0583). In the post-stress phase, both male *corticosterone* mice and *restrained* male mice significantly gained a comparable amount of weight [*corticosterone*: 1.9 (SE 0.3) g, *p* < 0.0001; *restrained*: 1.9 (SE 0.3) g, *p* < 0.0001]. Overall, the treatments led to significant differences in males (*p* < 0.0001) during the stress phase, with higher BWs in the corticosterone-treated group of 2.5 (SE 0.3) g. This effect was still pronounced in the post-stress phase with 2.5 (SE 0.3) g (*p* < 0.0001).

The estimated marginal means of BW of female mice in the baseline condition was similar for both treatment groups with *corticosterone* 22.0 (SE 0.2) g and *restrained* 22.2 g (SE 0.2) g (*p* = 0.9709). Corticosterone treatment in female mice led to a pronounced increase of 4.1 (SE 0.3) g (*p* = 0.0001) and a smaller increase in the restrained group of 0.9 (SE 0.3) g (*p* = 0.0217) in the stress phase. During the post-stress phase, the weight of the corticosterone group was significantly higher than in the stress phase with 2.2 g (SE 0.3) g (*p* < 0.0001) and for the restrained with 1.0 (SE 0.3) g (*p* = 0.0036). When comparing the different treatments in the stress phase, the female *corticosterone* group weighed 2.9 g (SE 0.3) (*p* < 0.0001) more than the female *restrained* and in the final post-stress phase, the difference was even more prominent with 4.2 g (SE 0.3 g) (*p* < 0.0001).

In comparison, males gained 15.9% and females 18.2% of total BW during corticosterone treatment in the stress phase. This indicates a higher sensitivity toward corticosterone treatment in female mice.

#### Burrowing Test

The overall estimate of burrowed material in Experiment 1 was 66.4% (SE 5.1%) (=intercept) (Figure 3B). We found a general difference due to treatment [treatment: *F*(1,383) = 11.7301, *p* = 0.0007] in the performance of the burrowing behavior. No general alterations due to sex or phase were detected. However, performance dropped significantly in the male *corticosterone* group from baseline 66.4% (SE 5.1) to 38.4% (SE 8.1) in the post-stress phase (*p* = 0.0441). All other groups showed no significant effects due to the phase.

In the baseline phase, no treatment effects were observed within the same sex. However, in the stress phase, male *corticosterone* burrowed 22.26% (SE 9.39) less than the *restrained* group (*p* = 0.0846). This effect was even more pronounced in the post-stress phase when the male *corticosterone* burrowed 40.00% (SE 11.50) less than the restrained (*p* = 0.0032). No effects became apparent for female comparisons of the treatment groups in all phases.

#### Fecal Corticosterone Metabolite Sampling

We observed the expected increase of FCM concentration in the *corticosterone*-treated mice (Figure 3C). The intercept was 56.2 (SE 25.0) ng/0.05 g. The concentration increased by 209.7 (SE 31.2) ng/0.05 g (*p* < 0.0001) compared to baseline 56.2 (SE 25.0) ng/0.05 g in male mice and 293.4 (SE 30.6) ng/0.05 g (*p* < 0.0001) in female mice [baseline phase 98.6 (SE 25.0) ng/0.05 g]. In the post-stress phase values normalized and decreased by 182.0 (SE 25.7) ng/0.05 g (*p* < 0.0001) and 200.2 (SE 26.5) ng/0.05 g (*p* < 0.0001), respectively. Post-stress values were not significantly different to baseline. Restraining did not alter FCM concentrations in our study.

#### Nest Test

Nesting scores were unaltered throughout the experiments (data not shown).

#### Time to Integrate Into Nest Test

The intercept for the latency to integrate the material into the nest site was 600.0 (SE 72.2) s (*p* < 0.0001). This was influenced by the treatment [*F*(1,206) = 16.75, *p* < 0.0001]. Overall, *restrained* mice were 197.3 (SE 102.1) s faster than the intercept (*p* = 0.055). We also found effects for phase [*F*(2,206) = 25.8974, *p* < 0.0001] and a treatment: phase interaction [*F*(2,206) = 3.64, *p* = 0.0278]. Again, no differences between the groups were found in the baseline phase. Neither did the treatment groups differ in the stress phase. We did see a general drop in latencies during the experiment. However, that was also visible in the control group. Additionally, in the post-stress phase, the latencies were significantly increased in the *corticosterone* groups in both male mice [196.6 (SE 52.9) s higher than *restrained*, *p* = 0.0015] and female mice [145.9 s (SE 52.9) higher than *restrained*, *p* = 0.0321]. Male mice tended to show higher latencies than females [sex: *F*(1,206) = 3.05, *p* = 0.0823].

#### Saccharin Preference

The mice showed altered preferences to saccharin drinking due to the treatment [treatment: *F*(1,220) = 7.28, *p* = 0.0074] and also in different experimental phases [phase: *F*(2,220) = 29.13, *p* < 0.0001], resulting in a significant interaction [treatment × phase *F*(2,220) = 21.3853, *p* = 3.259e−09] (Figure 3E). Overall the intercept was 88.2081% (SE 2.3136). Before the treatment, all groups showed similar preferences, but within the progression of the experiment, the preference developed differently for each treatment and sex [sex: *F*(1,220) = 33.30, *p* < 0.0001]. While there was a strong effect in *restrained* mice, the corticosterone treatment evoked no or only a mild effect. Male *corticosterone* mice showed no significant differences, neither between the baseline and the stress phase nor between the stress an the post-stress phase. Instead, an overall decrease from baseline [88.2% (SE 2.31) to post-stress (80.0% (SE 1.34) of 8.2% (SE 2.67), *p* = 0.0295] was visible. The female *corticosterone* mice showed very stable SP without differences between any phases. On the other hand, the *restrained* mice were highly affected by the treatment: male mice dropped from initially 93.5% (SE 2.31) by 21.43% (SE 2.83) (*p* < 0.0001) in the stress phase, female mice dropped from 92.8% (SE 2.31) by 14.0% (SE 2.83) (*p* < 0.0001). This was 11.3% (SE 2.31) and 11.9% (SE 2.31) less than in the male and female corticosterone groups. As soon as the treatment stopped, the preference for saccharin returned. The females showed no difference between baseline and post-stress preference. In males, the recovery was not as strong. Although the preference increased by 8.04% (SE 2.11) (*p* < 0.001) after the stress, the overall preference was only 84.8% (SE 1.34) and hence lower than before the treatment (*p* = 0.0165).

**FIGURE 4:**
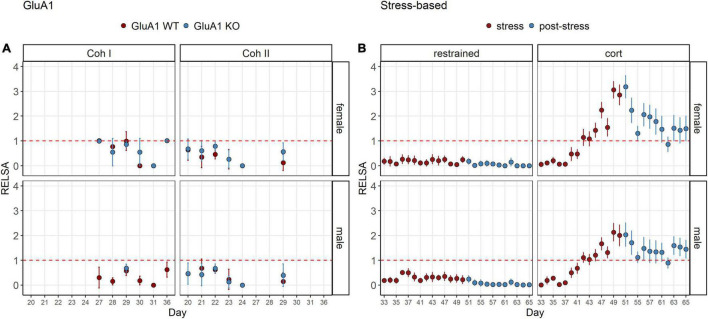
RELSA_flow_: comparison of all models in the RELSA in the course of the experiment. **(A)** The RELSA shows fluctuations throughout the assessment in both cohorts and sexes. **(B)** In the stress-based models, the increase of the RELSA during the stress phase is evident in the corticosterone group. In the post-stress phase, the values drop, but do not reach the initial level. Restraint treatment only evokes a minor effect in males on day 36, but females display no burden according to the RELSA. Restraint stress serves as reference. The RELSA includes body weight, burrowing performance and fecal corticosterone metabolite concentrations. The red line indicates the reference maximum. Data was analyzed using RELSA in R, GluA1 KO ♂ *n* = 11, ♀ *n* = 14, controls ♂ = 14, ♀ = 11, *n* = 10 per group in the stress-based experiment, except ♀ corticosterone *n* = 8, error bars are mean ± 95% CIs.

The RELSA_*flow*_ showed the severity over time (Figure 4). Although no stressful interventions were performed on the GluA1 KO mice, the severity was not as stable as in the *restrained* animals. Those showed nearly no effect even in the stress phase. There was only a very mild elevation on day 36. On the other hand, the *corticosterone* group revealed an increase that climaxed at the treatment’s end and then dropped. However, the RELSA score did not recover to the previous baseline on day 33. Still, it remained on a plateau above the score of 1, which equals the maximum severity of the restrained group.

**FIGURE 5:**
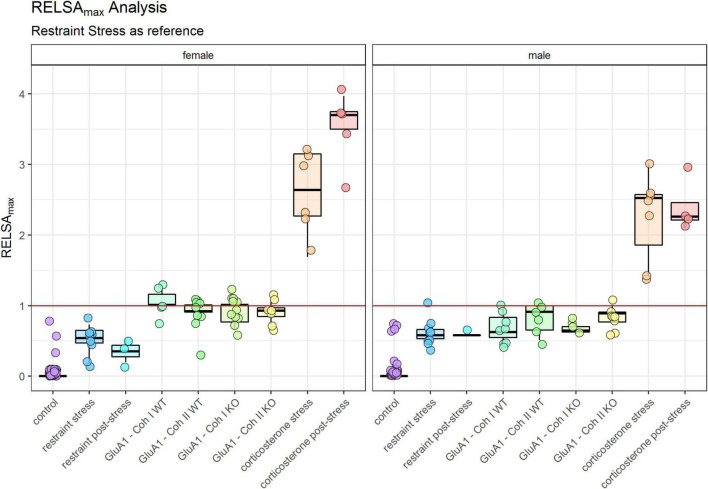
RELSA_max_: comparison of the maximum severity throughout the experiment detected using the RELSA score. Restraint stress serves as reference. In addition, the RELSA includes body weight, burrowing performance, and fecal corticosterone metabolite concentrations. The red line indicates the reference maximum. Data was analyzed using RELSA in R, GluA1 KO ♂ *n* = 11, ♀ *n* = 14, controls ♂ = 14, ♀ = 11, *n* = 10 per group in the stress-based experiment, except ♀ corticosterone *n* = 8. Data was analyzed using RELSA in R, GluA1 KO ♂ *n* = 11, ♀ *n* = 14, controls ♂ = 14, ♀ = 11, *n* = 10 per group in the stress-based experiment, except ♀ corticosterone *n* = 8.

The maximum severity throughout the experiment for each group is displayed in the RELSA_*max*_ (Figure 5). We found significant differences between male and female mice (Table 1). Even though the controls were only housed and received no specific treatment, some still displayed a deflection on the RELSA score at some time in the experiment. But in total, it was evident that this group served as a good baseline of severity. All other groups showed significantly higher RELSA scores – except the male mice in the post-restraint phase. We found more effects in female mice between the different models: GluA1 had higher scores than the *restrained* and the *corticosterone* groups higher scores than the *restrained* and GluA1 group. In males, we found no differences between GluA1 and *restrained*, but an elevation of *corticosterone* in the GluA1 groups.

**TABLE 1:** Results of statistical results from RELSA_*max*_.

Kruskal–Wallis					
	**Chi-squared**	**df**	***p***		
	
Females	241.8	8	<2.2e−16		
Males	99.101	8	<2.2e−16		
**Pairwise comparison (Wilcox-test, Holm corrected)**					

		***p***			

		**Females**		**Males**	

Control	Restraint stress	1.20E−33	****	4.20E−09	****
Control	Restraint post-stress	1.20E−21	****	n.s.	
Control	GluA1 – Coh I WT	1.40E−28	****	7.40E−09	****
Control	GluA1 – Coh II WT	1.50E−34	****	5.10E−08	****
Control	GluA1 – Coh I KO	8.90E−37	****	1.40E−05	****
Control	GluA1 – Coh II KO	2.90E−32	****	1.30E−09	****
Control	Corticosterone stress	1.30E−30	****	1.90E−06	****
Control	Corticosterone post-stress	1.40E−28	****	1.30E−06	****
Restraint stress	GluA1 – Coh I KO	6.60E−03	**	n.s.	
Restraint stress	GluA1 – Coh II KO	3.10E−02	*	n.s.	
Restraint stress	Corticosterone stress	4.20E−02	*	2.80E−01	*
GluA1 – Coh I WT	Corticosterone stress	n.s.		9.00E−03	**
GluA1 – Coh II WT	Corticosterone stress	4.20E−02	*	n.s.	
GluA1 – Coh I KO	Corticosterone stress	2.50E−02	*	n.s.	
GluA1 – Coh II KO	Corticosterone post-stress	4.40E−02	*	0.038	*

*p < 0.05, **p < 0.01, ***p < 0.0001.

## Discussion

This study aimed to determine the severity of several standard models for depression in mice and directly compare the relative burden introduced by different strategies, namely targeted mutagenesis of the depression-associated *Gria1* gene, stress-induction *via* subchronic oral corticosterone treatment, or temporary repetitive restraint. This is especially interesting as the various models will affect the subjects for different lifespans: while the GluA1 KO is lifelong and already relevant during development, the stress-based models can vary in duration and the animal’s age during onset. Since most depression-based animal experiments are performed in early adulthood, we compared the severity in this phase.

We assume that animals in depression models are affected negatively when compared to controls, even in a home cage-based analysis. As the strategies to induce depression in the animals were created to mimic the symptoms of depressive patients, it is plausible to assume that the well-being in such a model would be as impaired as it is in humans. Yet, the evaluation of suffering should be based on the animals’ state rather than anthropomorphic predictions. Hence, the severity assessment was based on established empirical measures of physiology and behavior (Bleich and Tolba, 2017; Moller et al., 2018; Koska et al., 2019; Seiffert et al., 2019; Bleich et al., 2020; Jirkof et al., 2020; van Dijk et al., 2020; Boldt et al., 2021; Buchecker et al., 2022). Furthermore, we have chosen to use parameters that hardly interfere with the animals and, therefore, should not confound the results by additional stress, e.g., more elaborate behavioral tests like tests for anxiety-related behavior. The behavioral parameters were derived from the natural behavioral repertoire of the mice in the home cage. In addition, these tests are all straightforward to perform, minimize the direct contact with the investigator and can be used by anybody who wants to assess the severity of their setups. In principle, this kind of evaluation is also possible simultaneously with experimentation due to the non-invasive nature of the tests.

### GluA1 KO Animals Show Impairment of Well-Being

We started our assessment with GluA1 KO mice and their littermate controls. GluA1 is a subunit of the AMPA subtype of glutamate receptors. Disturbances in the glutamate system are associated with psychiatric disorders, including mood disorders and schizophrenia (Pittenger et al., 2007; Sanacora et al., 2012; Eltokhi et al., 2020). Many studies have thoroughly described the mouse line used in this project. The loss of GluA1 leads to an increase in depression-associated learned helplessness (Chourbaji et al., 2008), hyperlocomotion in the open field (Wiedholz et al., 2008), novelty- and stress-induced locomotor hyperactivity, altered coping in the forced swim test and in approach-avoidance conflict tests (Fitzgerald et al., 2010), cognitive impairments in short-term memory, puzzle-solving and attention (Zamanillo et al., 1999; Bannerman et al., 2004; Sanderson et al., 2009; Ben Abdallah et al., 2011; Austen et al., 2021; Strickland et al., 2021). In addition, they show circadian disturbances, striking rest-activity pattern changes, and even reduced voluntary running (Ang et al., 2021), another valuable home cage-based parameter for well-being (Mallien et al., 2020; Weegh et al., 2020, 2021).

Some, but not all of these studies, also included female mice. We aimed to assess baseline severity in both sexes. We found the typical BW reduction in male GluA1 KO, not observed in females (Bannerman et al., 2004). The natural behavioral traits were compromised in GluA1 KO: the mice burrowed less, built less complex nests, and were slower in integrating new nesting material to their nest site. This indicates an impairment in well-being, which was cohort-dependent to some degree. One would assume that the same genotype would always create a similar burden on the animals. Instead, we found some effects only to be relevant for one cohort but not the other, sometimes even in a sex-dependent manner, e.g., the nesting of GluA1 KO females was only impaired in cohort II.

In contrast, nesting was impaired in both cohorts of males. This might be triggered by environmental factors or other differences in husbandry (Wurbel, 2017; Jirkof et al., 2020). FCM concentrations of baseline housing were comparable between KO and WT. Overall, one can see that the males’ and females’ burden is similar in the RELSA_*flow*_ (Figure 4) and RELSA_*max*_ (Figure 5). Moreover, although we found differences for some parameters, there is no observable difference between the KO and the WT in the RELSA.

The SP in GluA1 KO mice has been discussed previously and was not implemented in our current study. Austen et al. (2017) showed that GluA1 KO mice have a normal sucrose consumption level but reduced licking rates. Maksimovic et al. (2014) on the other hand, found increased sucrose preference compared to wild-type controls. As GluA1 KO mice often show hyperlocomotion, this preference might also be due to the need to compensate high energy. Strickland et al. (2021) even question whether GluA1 is implicated in the hedonic value at all.

The poor burrowing performance primarily drives the higher severity scores. Although mice were shown to consistently and vigorously burrow food pellets in the first 2 h of exposure (Deacon, 2009), we observed that the performance was delayed in our laboratory. Hence, we decided to observe the 6-h burrowing interval instead. In other studies, burrowing is even assessed overnight (Mallien et al., 2020). From our experience, mice that might not want to burrow after 2 h or even 6 h might have removed all the substrate from the burrowing tube 24 h later and even started building a nest in the tube, while others are not interacting with the burrowing substrate at all. However, the individual reduction of burrowing performance is generally considered an indicator of well-being impairments.

### Different Stressors Influence Well-Being Differently

We compared two stress-based strategies to elicit depression-associated symptoms in mice. The stress response is mediated by corticosterone, the major glucocorticoid expressed in rodents. Chronic corticosterone has been shown to induce anxiety- and depression-associated symptoms (Strekalova et al., 2004; Gourley et al., 2008; Mitra and Sapolsky, 2008; Zhao et al., 2008; Gourley and Taylor, 2009; Moda-Sava et al., 2019).

In the first approach, the animals are repetitively exposed to the stressful procedure of restraint, in the second approach, the corticosterone is not released physiologically in the mouse after a stressful experience but is instead provided in the drinking water. We used healthy wild-type mice in both strategies and observed their status before, during, and after the stress-based procedures.

The effects of daily *restraint* stress were mild. The BW stagnated during the stress procedure but normalized after the treatment phase in males and females. We also found no change in burrowing performance or TINT. Interestingly, the FCM concentrations did not rise due to the restraint. However, we did see a diminished preference for the saccharin solution in both sexes, from which the mice recovered in the post-stress phase. This indicates a strong emotional burden and a minor effect on physiological parameters. The restraint treatment hardly influences the RELSA. In the RELSA_*max*_, the restraint stress is the least severe treatment modality in the present study. We deliberately chose a mild restraint paradigm, with only 1 h per day and without an unpredictable or uncontrollable schedule – which is a vital factor in evoking depressive-like states in rodents (Vollmayr and Gass, 2013; Willner, 2017; Strekalova et al., 2022). This might also explain that the preference for saccharin only dropped to ∼75%. Longer restraint phases or unpredictability might increase this effect.

The response to the *corticosterone* treatment was very striking. We observed a rapid weight gain in male and female mice. Although BW alterations concerning animal well-being are typically associated with a loss, we considered this abnormal increase a burden. It is not surprising that the mice showed this increase, given that the treatment is also used to model obesity. However, studies in the literature using 0.1 mg/ml corticosterone in drinking water were only reported in males (Cassano et al., 2012). In addition, we observed untypical behavior in the home cage (limitation of movement, circling) in females after 18 days of treatment. In two cases, the behavioral alteration was so severe that we chose to euthanize the animals as they reached the humane endpoint. We then decided to shorten the procedure from the originally planned 21 days to avoid unnecessary suffering and further losses. The males displayed no such alterations. As soon as the treatment stopped, the BW normalized in both sexes. Of course, as expected, the FCM concentrations increased during the corticosterone treatment but normalized again at the termination of the treatment.

Male *corticosterone* mice showed several other well-being-associated deficits: their burrowing performance decreased during the stress phase. It did not recover during post-stress, their latency to integrate into the nest was increased post-stress, and their preference for saccharin solution was mildly reduced in the post-stress phase. While the *corticosterone*-treated mice in Moda-Sava et al. (2019) dropped from ∼80% preference to 60%, we only found a very mild effect: here, the preference dropped from 88% to only 80%. On the other hand, female corticosterone mice showed no SP or burrowing alterations. This is particularly interesting, as we detected the strong effects in the physiological FCM concentration and BW measures. This leads to the question: Which are the better indicators for the stress burden in this case: the parameters for the psychological or for the physiological burden? In the RELSA score, we used BW, burrowing performance, and FCMs to compare models as they were the most prominent parameters in our analysis. This results in very high RELSA scores in females. Based on this, using males for these specific approaches might be a relevant refinement measure. Especially since the females did not show anhedonic behavior at all.

Based on our data on the *restraint* and *corticosterone ingestion* approaches, we conclude that the *restraint* is a better and more refined model for depression: it evokes the depression-linked symptom of anhedonia with a generally lower burden, especially on the physiological level. In this case, choosing the *restraint* approach over *oral corticosterone* ingestion appears to be a possible refinement.

### Comparison of Burden: Stress vs. Genetic Evoked Model for Depression

The other critical observation in this study is comparing the burden of Experiment 1 and Experiment 2: stress-based and genetic alterations. The RELSA score allows the relative comparison of all assessed models. We cannot give an absolute estimate of the burden (mild, moderate, severe), but we can detect which treatment is the most or least detrimental. In the RELSA_*max*_ (Figure 5) the *corticosterone* treatment is the most severe model. All models show elevated RELSA compared to controls. There is no significant difference between the restraint and the GluA1 groups in males, and consequently, their burden is rated as similar. In females, there is a significant difference between the lower RELSA_*max*_ score of *restraint* stress to GluA1 KO but not to the GluA1 WT. Hence, the GluA1 KO is more severe in females than the daily restraining procedure, while there is no difference between the two procedures in males.

Given that GluA1 KO is likely to be a lifelong burden, while the effects of the stress-based treatments are mostly temporary, we assume that the overall burden is higher in the knockout model. This is not to say upcoming studies should avoid genetic manipulations: GluA1 KO mice show strong psychiatry-associated phenotypes and are an essential tool for gaining more insight into glutamatergic participation in the respective psychiatric disorders (Kilonzo et al., 2022). It is pivotal to have the opportunity to analyze the effects of genes on behavior. However, it is necessary to consider this burden in the harm-benefit analysis already in the beginning of upcoming projects. Genetic models can and must be justified (Wurbel, 2017; Grimm et al., 2019; Homberg et al., 2021). The researchers should be aware that the choice of a particular genotype might already be a choice against the well-being of the animals. With growing evidence for impaired well-being in genetic lines, researchers have to adjust to the new state of knowledge and accept that models, which formerly were not considered burdened, might be burdened after all. Additionally, the focus should be to alleviate the suffering as much as possible, e.g., taming the animals and avoiding anxiety.

### Anxiety in Severity Assessment

Anxiety constitutes a negative affective state and unpleasant feeling that often accompanies the anticipation of events. Of course, such a state impairs the well-being of the animals. Therefore, it is imperative to avoid anxiety in laboratory animals whenever possible. This is pivotal not only for their well-being, but also for the sake of data variability: gentle handling, e.g., cup or tunnel handling, have been shown to reduce anxiety as well as the variation of results at low costs (Hurst and West, 2010; Gouveia and Hurst, 2013, 2017, 2019; Henderson et al., 2020; Sensini et al., 2020). Even though anxiety is a known contributor to laboratory animal distress, we chose to not include it in this study. The assessed parameters were selected to be simple and accessible for everyone. Not every lab has the means and experience to assess anxiety. But also, testing outside the home cage can interfere with the treatment effects (Mallien et al., 2020). Transferring mice from one room to the other, introducing them into aversive environments, e.g., elevated mazes or brightly lit compartments, to evoke an approach-avoidance conflict would most likely cause an additional stress response – which we wanted to avoid in this baseline approach of severity assessment.

### Grading Severity Based on Evidence

According to EU directive 63/2010, all animal experiments have to be categorized by the expected degree of pain, suffering, distress, or lasting harm within the project. Concluding from our observations in this study, we cannot make an absolute assessment of severity. However, the animals recovered from the distress in the *restrained* group very quickly and did not show large deflections on the RELSA score. GluA1 KO was similar in males but elevated in females. In comparison with the *corticosterone* treatment, both models were much milder. Therefore, we would consider restraint treatment and GluA1 KO as moderate severity for the animals. Due to the high severity scores and the fact that we had to euthanize two mice in the *corticosterone* treatment group, we think that treatment with 0.1 mg/ml in drinking water for longer than 17 days might also be considered “severe,” at least in female mice. That is particularly interesting since other models, e.g., including foot shocks to induce learned helplessness, are considered “severe” according to the assignment criteria in annex VIII. Still, they show only minimal effects on home cage-based well-being parameters (Mallien et al., 2020). Based on these observations, learned helplessness would fall into the “moderate” category, especially since it is also a temporary effect.

In conclusion, we found welfare impairments in all addressed models of depression. This is not surprising as these models are used regularly to inflict symptoms of depression. It is essential to be aware of the burdens of the animals for regulatory and ethical reasons and choose the models wisely. The aim must always be on the benefit side of the harm-benefit balance, but acknowledging the damage and trying to reduce it, e.g., choosing the best model.

## Data Availability Statement

The original contributions presented in the study are included in the article, further inquiries can be directed to the corresponding author.

## Ethics Statement

The animal study was reviewed and approved by the Regierungspräsidium Karlsruhe.

## Author Contributions

AM, NP, CB, DI, RS, RP, ST, and PG: conceptualization and writing – review and editing. ST and AM: formal analysis and visualization. PG: funding acquisition. NP, CB, and AM: investigation. AM and PG: project administration. PG and RS: resources. AM, RP, and PG: supervision. AM, NP, CB, RS, RP, ST, and PG: validation. AM: roles/writing – original draft. All authors contributed to the article and approved the submitted version.

## Conflict of Interest

The authors declare that the research was conducted in the absence of any commercial or financial relationships that could be construed as a potential conflict of interest.

## Publisher’s Note

All claims expressed in this article are solely those of the authors and do not necessarily represent those of their affiliated organizations, or those of the publisher, the editors and the reviewers. Any product that may be evaluated in this article, or claim that may be made by its manufacturer, is not guaranteed or endorsed by the publisher.
